# Risk Assessment Profiles for Caregiver Burden in Family Caregivers of Persons Living with Alzheimer’s Disease: An Exploratory Study with Machine Learning

**DOI:** 10.3390/ejihpe15030041

**Published:** 2025-03-20

**Authors:** Laura Brito, Beatriz Cepa, Cláudia Brito, Ângela Leite, M. Graça Pereira

**Affiliations:** 1 Psychology Research Centre, School of Psychology, University of Minho, 4710-057 Braga, Portugal; britolaura@hotmail.com; 2 INESC TEC, 4200-465 Porto, Portugal; beatriz.cepa@inesctec.pt (B.C.); claudia.v.brito@inesctec.pt (C.B.); 3 Department of Informatics, University of Minho, 4710-057 Braga, Portugal; 4 Centre for Philosophical and Humanistic Studies, Portuguese Catholic University, 4710-362 Braga, Portugal; aleite@ucp.pt

**Keywords:** distress, family stress, forgiveness, heart rate variability, predictive models

## Abstract

Alzheimer’s disease (AD) places a profound global challenge, driven by its escalating prevalence and the multifaceted strain it places on individuals, families, and societies. Family caregivers (FCs), who are pivotal in supporting family members with AD, frequently endure substantial emotional, physical, and psychological demands. To better understand the determinants of family caregiving strain, this study employed machine learning (ML) to develop predictive models identifying factors that contribute to caregiver burden over time. Participants were evaluated across sociodemographic clinical, psychophysiological, and psychological domains at baseline (T1; *N* = 130), six months (T2; *N* = 114), and twelve months (T3; *N* = 92). Results revealed three distinct risk profiles, with the first focusing on T2 data, highlighting the importance of distress, forgiveness, age, and heart rate variability. The second profile integrated T1 and T2 data, emphasizing additional factors like family stress. The third profile combined T1 and T2 data with sociodemographic and clinical features, underscoring the importance of both assessment moments on distress at T2 and forgiveness at T1 and T2, as well as family stress at T1. By employing computational methods, this research uncovers nuanced patterns in caregiver burden that conventional statistical approaches might overlook. Key drivers include psychological factors (distress, forgiveness), physiological markers (heart rate variability), contextual stressors (familial dynamics, sociodemographic disparities). The insights revealed enable early identification of FCs at higher risk of burden, paving the way for personalized interventions. Such strategies are urgently needed as AD rates rise globally, underscoring the imperative to safeguard both patients and the caregivers who support them.

## 1. Introduction

Dementia, especially Alzheimer’s disease (AD) creates significant global challenges, particularly in countries with high life expectancies (Meza-Cely et al., 2020). Over the past three decades, the prevalence of Alzheimer’s and other dementias has soared, with associated deaths increasing substantially. In Portugal, an estimated 153,000 people live with dementia, including 90,000 with Alzheimer’s (Organization for Economic Co-Operation and Development [OECD], 2021).

Dementia involves cognitive decline and dependency, affecting emotional well-being (Bessey & Walaszek, 2019). Many individuals living with AD require a family caregiver, usually an unpaid partner, relative, or friend (Manevich et al., 2023). Family caregivers (FCs) are often women, middle-aged, unemployed, or domestic workers who may have their health issues (Ruisoto et al., 2020).

Several studies indicate that FCs face higher risks of physical and mental health issues as they grapple with emotions such as guilt, resentment, and sadness, along with increased distress, family stress, and burden (Hellis & Mukaetova-Ladinska, 2023; Lindeza et al., 2020) associated with a reduced quality of life (Loo et al., 2022). However, while the previous studies highlight important findings, it remains crucial to understand why these factors matter and how they interconnect to impact a caregiver’s well-being.

Recent research has highlighted the profound impact of family stress on caregivers’ well-being. Irregular or insufficient care assistance has been associated with more burden on caregivers (Brown et al., 2020). During COVID-19, the suspension of formal assistance and care center closure further increased FCs’ responsibilities (Canevelli et al., 2020), with an impact on stress levels, conflict, loneliness, and overall quality of life (Greenberg et al., 2020).

The caregiving literature reveals a balanced perspective, acknowledging both positive and negative aspects (Costa et al., 2021; Quinn et al., 2019). Positive experiences, including personal capabilities, competence in caregiving, and strengthened relationships, have been associated with less caregiver burden (Abulaiti et al., 2022; Nemcikova et al., 2023).

While the existing literature has explored several psychological, social, and clinical factors contributing to caregiver burden, a better understanding of how specific psychophysiological markers and coping mechanisms play a role in this dynamic is needed. Heart Rate Variability (HRV) is a key psychophysiological marker of autonomic nervous system functioning and stress regulation. FCs’ burden has been associated with chronic stress, which can be reflected in lower HRV, indicating reduced physiological resilience (James et al., 2021). Understanding HRV, in caregivers, may provide important insights into stress-related health risks and potential intervention targets (Kim et al., 2018).

Forgiveness has been associated with better emotional regulation and improved relationships in stressful family dynamics (Kaleta & Mróz, 2020). In FCs, forgiveness may serve as a coping mechanism to help reduce marital distress and family conflict, which are significant burden contributors (Rasmussen et al., 2019). Studies specifically focusing on the caregivers of persons living with AD have highlighted forgiveness as an important factor in adjusting to the challenges of caregiving (McGee et al., 2021). Additionally, Bintamur (2020) found forgiveness to be inversely correlated with burden, suggesting its positive impact on well-being.

In AD, caregivers’ subjective burden is influenced by factors such as age, sex, spousal’s relationship, unemployment, and health issues (Kong et al., 2021), as well as caregiving intensity, patient frailty, and dementia severity (Vrettos et al., 2023). Gaspar et al. (2023) highlighted female gender, older age, lack of employment, and higher education as key contributors. Also, the mental functioning of the person living with AD, caregiver’s age, and having limited space at home predicted the caregivers’ burden (Loo et al., 2022; Tulek et al., 2020). Financial strain and organizational support needs were associated with a higher burden (So et al., 2021), with female caregivers experiencing higher distress, especially during the pandemic (Zwar et al., 2023).

Machine learning (ML) offers accurate predictions by capturing complex relationships and handling large datasets (Rosenbusch et al., 2021), enabling a deeper understanding of the factors influencing caregiver burden. As a result, more accurate predictions may be achieved that will inform the development of targeted interventions to support FCs, particularly for those at a high risk of experiencing psychological distress.

This study is grounded in Pearlin et al.’s (1990) model, which provides a comprehensive framework for examining several factors contributing to the caregiver‘s burden. The model includes background and context variables (sociodemographic characteristics), primary stressors (e.g., dementia clinical severity, time spent in caring, care duration, etc.), secondary stressors (e.g., family stress, distress, caregiving competence, personal gains, and HRV), and mediators (e.g., health behaviors and forgiveness) that predict burden. HRV was included as a psychophysiological indicator of autonomic stress regulation, given its association with the caregiver’s well-being. Forgiveness was selected as a psychological factor influencing emotional resilience and a coping strategy to manage caregiving-related stress. While many studies have used Pearlin’s model to examine caregiver burden, traditional statistical methods may struggle to capture the full interactions between sociodemographic, clinical, psychological, and physiological factors. Incorporating ML techniques offers an innovative way to understand this complex relationship between multiple burden-related variables and their interaction over time.

The purpose of this study was to identify burden risk profiles using ML with Decision Tree algorithms, considering sociodemographic, clinical, psychophysiological, and psychological variables at T1 (baseline), T2 (six months later), and T3 (six months after T2).

## 2. Materials and Methods

### 2.1. Ethics, Procedures, and Sampling

This study followed the ethical guidelines of the Declaration of Helsinki and was approved by the Ethics Committee of a major university in the North of Portugal (Ref. CEICSH 46/2020). FCs were recruited from the IADem Plan, a support program for persons living with AD, in Northern Portugal.

Inclusion criteria were as follows: being 18 or older, caring for a family member with AD, and using the Portuguese National Healthcare System. Exclusion criteria included formal caregivers, those receiving psychological support, or those with cognitive deficits. A total of 175 participants were screened, and 45 were excluded (37 based on criteria, 8 declined). At T1, 130 participants were included. At T2, 15 were no longer caregivers, and 1 had moved to a nursing home, leaving 114 participants. At T3, 14 more participants stopped caregiving and 8 entered nursing homes, resulting in a final sample of 92. Data were collected at T1 (*N* = 130), T2 (*N* = 114), and T3 (*N* = 92) over 12 months (2021–2022).

Data collection occurred in participants’ homes, during COVID-19 lockdowns. Baseline assessments included sociodemographic and clinical data, distress, burden, health behaviors, forgiveness, caregiving competence, personal gains, family stress, and HRV. At T2 and T3, participants completed the same T1 assessments. The burden, at T3, was the outcome variable. AD and frailty were assessed for inclusion in the study with the diagnosis confirmed by the patient’s neurologist (provided by IADem Plan). Cognitive assessments and clinical evaluations ensured all participants met the diagnostic criteria. Each assessment took approximately 45 min to complete and was administered in an interview format. Participants had a prior relationship with the researchers, in the context of home visits conducted by a team of two trained researchers, who followed a standardized protocol.

### 2.2. Instruments

The sociodemographic and clinical questionnaire assessed sociodemographic variables (age, gender, education, professional status, and marital status), caregiver relationship (duration and hours of care), and clinical variables for both caregivers (support received and chronic illness) and persons living with AD (substance use, memory issues, and neurological/psychiatric conditions).

**Clinical Dementia Rating** (CDR), (Morris, 1993): This scale evaluates cognitive impairment across six domains: memory, orientation, judgment, community affairs, home and hobbies, and personal care. Each domain is rated on a 5-point scale ranging from 0 (no cognitive impairment), 0.5 (questionable dementia), 1 (mild cognitive impairment), 2 (moderate cognitive impairment), and 3 (severe impairment). In this study, AD severity was categorized into three stages: mild, moderate, and severe.

**Zarit Burden Interview Scale** (ZBI), (Zarit & Zarit, 1983): This scale measures perceived burden in FCs, in domains such as health, social and personal life, finances, emotions, and relationships. Higher scores indicate a higher burden. Cut-off points include: no overload (below 46); mild overload (46–56); and severe overload (above 56). The original version showed a Cronbach’s alpha of 0.93, and 0.94, in the present study.

**Caregiving Competence Scale** (CCS), (Pearlin et al., 1990): This scale evaluates caregivers’ perceived competence in providing care. Scores range from 4 to 16, with higher scores indicating greater care competence. The original scale showed good internal consistency (α = 0.74), and in this study, Cronbach’s alpha was 0.93.

**Personal Gain in Caregiving Scale** (PGSS), (Pearlin et al., 1990): This instrument assesses the personal growth experienced by caregivers while navigating the challenges of caregiving. With strong internal consistency (Cronbach’s α = 0.76), scores on the 4-item subscale range from 4 to 16, with higher scores indicating greater personal growth. In our study, Cronbach’s alpha for this scale was 0.92.

**Index of Family Relations** (IFR), (Hudson, 1993): This instrument assesses family stress, reflecting the severity of relational problems. Scores range from 0 to 100, with higher scores indicating greater family stress. Two cut-off points are defined: below 30 signifies no family stress, while 70 or higher indicates severe stress. The scale reveals alpha values of 0.95 in the original version, and 0.91 in our study.

**Health Behaviors for Informal Caregivers Scale** (HBIC), (GISEF, 2023): This scale was adapted from a general lifestyle scale (Pereira & Pedras, 2009) assessing protective and risky health behaviors and validated in caregivers of persons living with AD. The adapted version includes nine items assessed on a four-point Likert scale, with higher scores indicating a healthier lifestyle. In this study, the alpha for the nine items was 0.71.

**Heartland Forgiveness Scale** (HFS), (Thompson et al., 2005): The HFS is an 18-item self-report questionnaire that measures the tendency to forgive. Scores range from 18 to 126, with higher scores indicating more forgiving. The original scale presented a Cronbach’s alpha of 0.86 and in the current study was 0.85.

**Depression Anxiety and Stress Scale** (DASS-21), (Lovibond & Lovibond, 1995): The DASS-21 assesses distress. Scores range from 0 to 63, with higher scores indicating more distress. The original DASS-21 showed a Cronbach’s alpha of 0.93 (Henry & Crawford, 2005), and was 0.97, in the present study.

**Heart Rate Variability** (HRV), (Elite HRV, 2019): The CorSense device by Elite HRV provides an objective measure of stress, comparable in accuracy to a 5-lead ECG/EKG. HRV sessions recorded through the CorSense mobile application last five minutes to ensure stability in results. Higher HRV scores indicate lower stress levels. The assessment involves capturing R-R intervals, calculating RMSSD, and applying natural log (ln). The resulting HRV score ranges from 0 to 100, with standard values provided for different age and gender groups: age 65–75 years (men): mean = 52.66, SD = 12.70; age 65–75 years (women): mean = 49.35, SD = 11.10. 

### 2.3. Data Analysis

Initially, descriptive statistics were used to characterize the sample and describe the data’s basic features using SPSS (Statistical Package for the Social Sciences), version 28.0 (IBM, Armonk, NY, USA).

To investigate potential differences between participants who dropped out of the study and those who remained a binary logistic regression analysis was performed. The dependent variable was dropout status at any assessment point (1 = remained in the study; 0 = dropped out), while the independent variables included FCs’ characteristics. The model’s overall fit was assessed using the Omnibus Tests of Model Coefficients, and its performance was evaluated through metrics such as Nagelkerke *R*^2^ and classification accuracy.

The results indicated that the model was not statistically significant (χ^2^ = 12.290, *p* = 0.197), and none of the predictor variables reached statistical significance (*p* > 0.05). The model’s overall classification accuracy was 73.8%, suggesting that FCs’ characteristics were not significantly associated with study dropout.

A second binary logistic regression was conducted to examine potential differences based on the characteristics of persons living with AD. The dependent variable remained dropout status, while the independent variables included gender, age, marital status, years of education, onset of memory problems, and prior treatments for memory issues. Similarly, to the first model, the results showed no statistical significance (χ^2^ = 7.968, *p* = 0.240), with none of the predictor variables reaching significance (*p* > 0.05). The model’s overall classification accuracy was 71.5%, indicating that the characteristics of persons living with AD were not predictive of dropout.

ML algorithms were used since they model complex, nonlinear relationships, capturing interactions between multiple predictors and improving prediction accuracy over time. For longitudinal studies, several ML approaches may be leveraged, such as Decision Trees, Random Forest, and Gradient Boosting Machines (GBMs), which were used in the present study (Witten et al., 2011).

Decision Trees are used for classification and regression, navigating from root to leaf nodes based on features to make predictions. Random Forest, an ensemble of Decision Trees, mitigates overfitting by aggregating predictions, with the number of trees and maximum depth parameters influencing performance and computational burden. Gradient Boosting Machine (GBM) algorithms like XGBoost, LightGBM, and CatBoost use Decision Trees as base learners. XGBoost optimizes speed and performance through error correction and regularization. LightGBM, designed for larger datasets, employs histogram-based splitting and customizable loss functions. CatBoost focuses on categorical data, employing techniques like ordered boosting to enhance learning by considering feature ordering. These algorithms sequentially add weaker learners to correct the mistakes of previous ones. XGBoost, LightGBM, and CatBoost enhance performance through speed optimization, regularization, and handling categorical data efficiently, with each offering unique features for improved learning. 

The ML algorithms described represent state-of-the-art approaches to handling complex data, leveraging Decision Trees as foundational components in ensemble learning and were used in the present study to train a risk assessment profile to identify the caregiver’s burden, tackling a regression-based problem. Knowing which variables impact FC’s burden and which do not, will add important information from a heuristic point of view. Therefore, a thorough evaluation was performed using different ML algorithms. Five algorithms were tested to find a correlation between the longitudinal data collected at the specified time-points. The best results showed a correlation (*R*^2^) of up to 0.70 with caregiver’s burden.

### 2.4. Pre-Processing

The goal of the pre-processing stage was to remove any missing values and group the features (i.e., the collected variables) into sets to be used in the training stage. The first objective was to remove the null data points, meaning that any individual who could not be evaluated in the three time-points was discarded. The most relevant features identified to analyze correlations were as follows: the severity of AD, HRV, caregiving competence, personal gain, distress, family stress, and forgiveness was assessed through questionnaires as well as the caregiver’s age, sex, marital status, education, duration of care provided, and the presence of chronic disease in the caregiver. Additionally, the age, sex, marital status, and education of the person living with AD were also included, along with the onset of memory problems and any family history of neurological or psychiatric conditions.

For the target output (burden), the data were organized into multiple datasets with the following principles: the columns related to other outputs were also removed during the pre-processing stage, as it was not desirable to use potential output classes as input for classification for any target output class. The features were then grouped as follows: each set of data corresponding to a different evaluation time-point (T1 and T2) resulted in all “T1_” features which were grouped into T1 and all “T2_” features were grouped into T2. Furthermore, those features that are constant and not specific to a particular time-point (e.g., sex, age, education, marital status, duration of care, presence of chronic diseases in family caregiver, presence of dementia in the family, starting of AD, and clinical dementia rating) were grouped as “baseline”.

All the groups were combined during the training stage to understand their impact on burden prediction. A total of six distinct datasets were used in the present study: T1 and T2 with only the changing features (named T1 and T2), T1 and T2 with all the features (named T1_base and T2_base), a combination of datasets T1 and T2 (named T1_T2), and a combination of datasets T1_base and T2_base (named T1_T2_base).

### 2.5. Training

Each one of the algorithms was trained and tested with the previously created datasets and optimized accordingly. The training of each algorithm was carried out under percentage split and cross-validation. The first mechanism split the dataset into training and testing datasets at a percentage of 80/20 (train/test).

The cross-validation (CV) pattern used the full dataset for training and testing during several iterations. The dataset was split into folds, and for each training iteration was tested with a different fold. In this methodology, we used a CV of 10-fold. The results depicted the best results for any of the techniques. Moreover, all the Scikit-Learn and GBM algorithms resort to GridSearchCV. This optimization allows findings to have the best parameters (i.e., tree depth, the number of iterations, or the number of estimators) for each model without the need to strictly define them initially, promoting the usage of the best possible model in each different execution.

When dealing with non-categorical data such as the ones in this study, where the target output can be depicted from a scale of 0 to 105, the best metrics to evaluate the model’s performance are based on the Root Mean Squared Error (*RMSE*) and the correlation of determination, *R*^2^. In detail, the *RMSE* identifies the root of the average of the squared differences between the predicted and the actual values.

All the experiments were executed on Visual Studio Code in MacOS Sonoma 14.2.1. The following methodology was implemented in Python 3.11, using the libraries NumPy (v1.19.5), and Pandas (v1.3.5) and for the ML algorithms, we applied both the Scikit-Learn (v1.0.2) library and the Weka framework.

## 3. Results

Table 1 summarizes the characteristics of the 130 FCs, who were predominantly female (77.7%) and spouses (44.6%) or daughters (42.3%), with a mean age of 66 years old. Most FCs resided in rural areas, had lower education levels, and often shared caregiving duties with at least one additional caregiver. Among the persons living with AD, 43 (33.1%) were in the mild stage, 37 (28.5%) were in the moderate stage, and 50 (38.5%) were in the severe stage.

### Burden Profiles

Table 2 displays the initial profiles. For the burden profiles, data from T1, T1 and T2 (T1_T2) evaluations were used. All datasets were included in the table since base features are also relevant for the final profiles regarding the burden prognosis prediction profiles. The initial evaluation of multiple models aimed to identify those with satisfactory performance, and a threshold of 0.5 (*R*^2^) was chosen for each model selection. Decision Trees in Scikit-Learn yielded correlation values above the 0.5 threshold, indicating their effectiveness in predicting burden.

**Figure 1:**
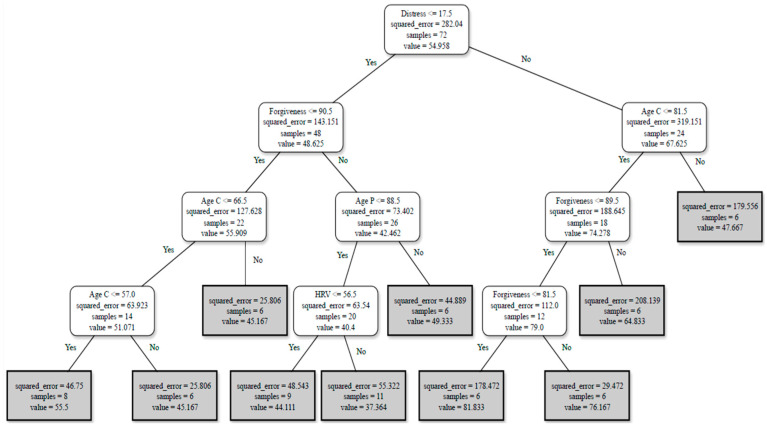
Profile 1—T2 Base.

Based on the initial findings, three primary profiles were developed, primarily relying on the results obtained from the Decision Trees. Validation across several algorithms confirmed the importance of the selected dataset. Results indicated that the data at T2 with baseline features held the most relevance, reducing *RMSE* values and increasing data correlation. Consequently, the first profile (Figure 1) was based solely on T2_base data, encompassing variables exclusively from T2 to develop a risk assessment profile for understanding FCs burden. This profile revealed three rules derived by tracing a full path from the root node to each leaf node. Each rule carried its probability of accuracy, determined by the number of samples of the leaf node and the dataset used to generate the profile. The rules were as follows:

1. For FCs reporting ≤ 17.5 on distress scores and ≥90.5 in forgiveness at T2, with an FCs’ age ≤ 66.5 or ≤57, the burden score perception will be around 51.
2. For FCs reporting ≤ 17.5 on distress scores and ≥90.5 in forgiveness at T2, with the person living with AD’s age ≤ 88.5 and HRV ≥ 56.5, the burden score perception will be around 40.
3. For FCs reporting >17.5 on distress scores, with age ≤ 81.5 and forgiveness at T2 ≤ 89.5 or ≤81.5, the burden score perception will be around 79.

**Figure 2:**
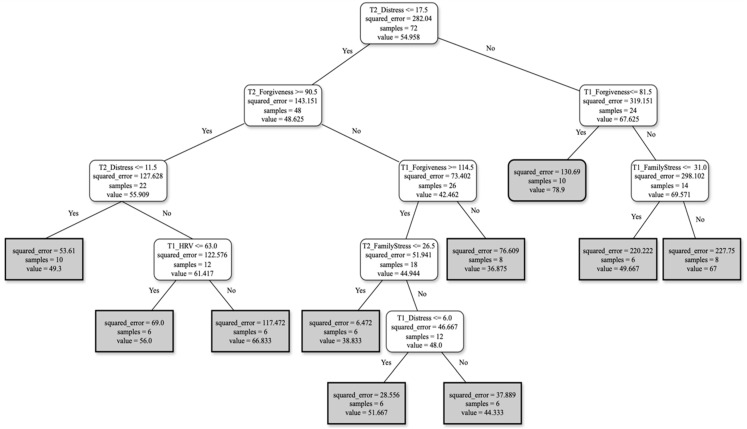
Profile 2—T1_T2.

A second profile was found based on T1 and T2 features (Figure 2). Based on this profile, three different rules were extracted:

1. For FCs reporting distress ≤ 17.5 and forgiveness ≥ 90.5 at T2, with distress ≤ 11.5 and HRV ≤ 63 at T1, the burden score perception will be around 61.
2. For FCs reporting distress ≤ 17.5 and forgiveness ≥ 90.5 at T2, with forgiveness at T1 ≥ 114.5, family stress at T2 ≤ 26.5 and distress ≤ 6 at T1, the burden score perception will be around 48.
3. FCs reporting distress ≥ 17.5 at T2, with forgiveness ≤ 81.5 at T1 and family stress at T1 ≥ 31, will show a burden score perception of around 69.

**Figure 3:**
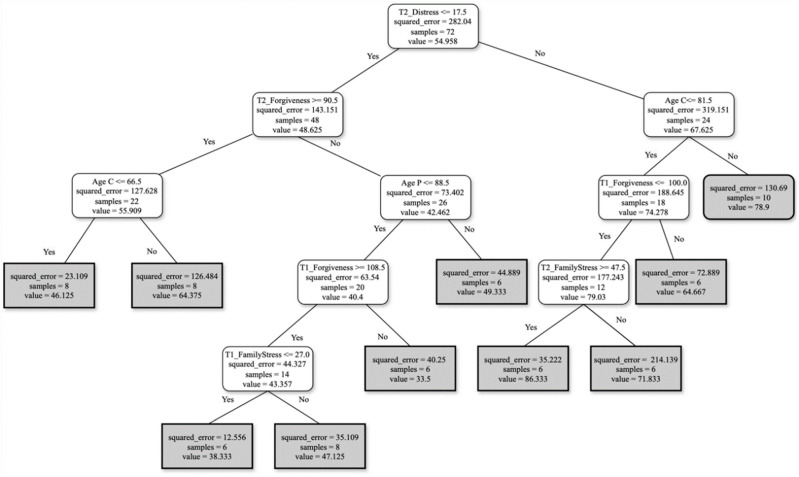
Profile 3—T1_T2_Base.

A third profile was found based on T1 and T2 features combined with the data base set (Figure 3) to enhance changes over time in the psychological variables considering sociodemographic and clinical features. Based on this profile, three different rules were extracted:

1. For FCs reporting distress ≤ 17.5 and forgiveness ≥ 90.5 at T2, with the FC’s age ≤ 66.5, the burden score perception will be around 56.
2. For FCs reporting distress ≤ 17.5 and forgiveness ≥ 90.5 at T2, with person living with AD’s age ≤ 88.5, and finally, forgiveness ≥ 108.5 and family stress ≤ 27 at T1, the burden score perception will be around 43.
3. For FCs reporting distress ≥ 17.5, with FC’s age ≤ 81.5, forgiveness at T1 ≤ 100, and family stress, at T2 ≥ 47.5, the burden score perception will be around 79.

## 4. Discussion

This study employed machine learning (ML) to identify key predictors of caregiver burden through predictive modeling, offering deeper insights into the complex dynamics driving this challenge. Results showed that FCs were predominantly female (77.7%), mostly spouses (44.6%) or daughters (42.3%), with an average age of 66, from rural areas, and lower education levels (M = 4.53 years, SD = 3.58). Some participants shared caregiving responsibilities. These findings align with the existing literature on the prevalence of women, spouses, and daughters in caregiving roles (Damian et al., 2023).

The exploration of burden profiles included several datasets and algorithms to identify three distinct profiles through the Decision Trees, each offering insights into different aspects of caregiver burden perception. The three profiles, identified in the present study, underscore the complexity of caregiving for persons living with AD, revealing the emotional, physical, and financial challenges involved. Caregivers invest substantial time and effort in providing support, often facing numerous stressors and burdens in the caregiving process.

This identification of the three distinct caregiver burden profiles, were shaped by several emotional, physiological, and demographic factors. The first profile featured FCs with low distress, high forgiveness, and higher HRV, which were linked to lower burden. The second profile highlighted FCs with elevated distress, lower forgiveness, and high family stress, associated with severe burden. The third profile integrated longitudinal data with sociodemographic factors, revealing how changes in distress, forgiveness, and family stress influenced burden over time. This second profile showed that lower distress at T2, higher forgiveness (T1), reduced family stress (T1), and a younger age of the person living with AD were all associated with lower burden. These findings underscore the critical role of emotional resilience, stress, and caregiver characteristics in shaping burden levels. According to the first predictive model, FCs experiencing low distress (≤17.5 scores) and higher forgiveness (≥90.5) at T2, while caring for persons living with AD less than 88.5 years old with HRV ≥ 56.5, showed no burden. This outcome emphasizes prior research outcomes indicating that FCs with lower distress levels and higher forgiveness may cope better and perceive their caregiving role as less burdensome (Levy et al., 2021; McGee et al., 2021). The capacity to forgive appears crucial, as those who forgive more tend to experience lower burden levels, emphasizing its role as a coping mechanism throughout the caregiving journey (López et al., 2021; Strelan, 2020). Moreover, this profile underscores the influence of emotional resilience and physiological well-being on the caregiver’s burden. Higher HRV, adjusted for age, may indicate improved autonomic nervous system function, better cardiovascular health, and lower stress levels, resulting in a reduced perceived burden (James et al., 2021).

Conversely, FCs experiencing higher distress levels (≥17.5), aged ≤ 81.5 years, and lower forgiveness (≤89.5) exhibited severe burden. This outcome is in accordance with previous research indicating that FCs with elevated distress and diminished forgiveness tend to perceive their caregiving role as more burdensome (Kılıç et al., 2024; Strelan, 2020).

The inclusion of age and forgiveness criteria underscored the intricate interplay between emotional states and the caregiver’s characteristics in shaping burden highlighting the importance of age on caregivers’ experiences and burden perceptions. While the age difference may have not been substantial, nonetheless, younger caregivers may face distinct challenges or stressors contributing to more burden. This outcome underscores the need for tailored support interventions for younger caregivers to address their unique needs. Younger caregivers may feel the impact of caregiving on their daily lives more, particularly in recreational and social activities, especially when there are no respite care opportunities (Häikiö et al., 2020). The COVID-19 pandemic also exacerbated the caregiving burden, with a significant portion of FCs reporting increased strain, isolation, and family strain (Tulek et al., 2020). Additionally, juggling caregiving responsibilities with employment, education, or childcare, is more problematic in younger FCs, which may result in increased stress and perceived burden (Tsai et al., 2021).

Expanding beyond time-point T2, the second profile integrated features from both T1 and T2 (Figure 2), providing a more global perspective on family caregiver burden dynamics. The rules derived from this profile reveal complex relationships among distress, forgiveness, family stress, HRV, and age over different time-points.

FCs exhibiting low distress at both T1 (≤6) and T2 (≤17.5), higher forgiveness at both T1 (≥114.5) and T2 (≥90.5), with reduced family stress at T2 (≤26.5) reported lower burden. This finding highlights the impact of forgiveness and family stress on caregiver burden. FCs who displayed more forgiveness and lower family stress tend to perceive their caregiving role as less burdensome (Nemcikova et al., 2023; Sołtys & Tyburski, 2020).

Despite the challenges, FCs often derive fulfillment and purpose from their role, finding satisfaction in making a positive impact on their loved one’s life (Quinn et al., 2019). This positive relationship dynamic fosters mutual trust, empathy, and effective communication, enabling FCs to navigate their responsibilities more effectively (Gräler et al., 2022). Adopting forgiveness, recognizing the positive aspects of caregiving, and receiving support from family members may mitigate the sense of burden and enhance caregivers’ quality of life over time (Nemcikova et al., 2023; Sołtys & Tyburski, 2020).

FCs experiencing elevated distress at T2 (≥17.5), lower forgiveness (≤81.5), and heightened family stress (≥31) at T1 faced severe burden (Lindeza et al., 2020). These stressors encompass interpersonal conflicts, financial strains, and emotional burdens within the family unit, exacerbating caregivers’ mental distress (Fagerström et al., 2020; Gaspar et al., 2023). Emotional burdens stemming from witnessing the decline of a loved one with dementia exacerbate caregivers’ mental distress.

The third profile integrated data from both T1 and T2, along with sociodemographic and clinical features, enriching the analysis (Levy et al., 2021; McGee et al., 2021). By considering a broader range of variables, this profile revealed complex associations between distress, forgiveness, family stress, and age, providing a more complex picture of the predictors of burden perception.

FCs reporting low distress at T2 (≤17.5) and higher forgiveness at T1 (≥108.5) and T2 (≥90.5), caring for a person living with AD aged ≤88.5 years old, and lower family stress at T1 (≤27) showed lower burden. This finding highlights the nuanced relationship between FC’s burden and factors such as age and forgiveness (Allen et al., 2020). While the relationship between the person with AD’s age and FCs’ burden may not be immediately intuitive, it could reflect the challenges associated with increased care needs due to health complications. 

FCs reporting higher distress levels (≥17.5) at T2, younger age (<81.5 years), lower forgiveness at T1 (≤100), and higher family stress at T2 (≥47.5) showed severe burden. This finding highlights the significant role of distress, forgiveness, and family stress in shaping caregiver burden perception (Fagerström et al., 2020; Gaspar et al., 2023).

The present study provides a novel contribution by showing how psychophysiological indicators, such as HRV, interact with psychological factors like distress and forgiveness to influence caregiver burden. Unlike previous studies that focused primarily on emotional or social factors, the findings also incorporated a stress biomarker to identify distinct burden profiles. Additionally, the stability of forgiveness as a key predictor of long-term burden perception, and the interaction between FCs and the characteristics of the person living with AD, also shaped the level of burden experienced. The integration of psychophysiological and psychosocial factors offers a more comprehensive understanding of FCs’ stress and opens new avenues for targeted interventions.

## 5. Limitations

This study is the first to use Decision Tree algorithms to establish risk assessment profiles for caregiver burden in those caring for persons with AD. The identified profiles offer valuable insights based on demographic, clinical, and caregiving-related factors, independent of dementia severity. Additionally, the findings highlight the potential of ML algorithms for analyzing longitudinal data to uncover patterns and relationships among variables. Furthermore, the results showed enormous potential for leveraging ML algorithms for longitudinal data to understand patterns and associations. Nonetheless, this study presents some limitations, such as the data sample size, requiring a larger cohort to replicate the results in a future study. Data imputation and oversampling mechanisms (e.g., multiple imputation and synthetic minority oversampling) are considered potential techniques to enhance the robustness of the dataset but were not implemented in the current. Feature selection techniques, such as lasso regression, could further identify the most relevant features to improve model accuracy and generalizability. Additionally, other state-of-the-art algorithms, such as support vector machines, neural networks, and elastic net regression, could also be implemented to understand their individual contribution, as well as their combination within an ensemble methodology.

Although ML models can be applied to small datasets, the variability in sample size across profiles (ranging from seventy-two participants to as low as six) introduces challenges in model reliability. The minimum sample size required for machine learning depends on the number of features and model complexity. For Decision Tree algorithms, a general guideline is that the sample size should be at least 10 times the number of features to avoid overfitting (Rosenbusch et al., 2021). While our results provide valuable insights, future studies should use larger and more balanced samples to ensure the robustness and reproducibility of the predictive models. Future selection techniques, such as lasso regression, to further identify the most relevant features, data imputation, and oversampling mechanisms (e.g., multiple imputation, synthetic minority oversampling) could help create a more robust dataset and, consequently, a better model with improved predictive performance and generalizability. Additionally, other state-of-the-art algorithms, such as support vector machines, neural networks, and elastic net regression, could also be implemented to understand how they would deal with these datasets, as well as the combination of them, within the ensemble methodology. Although acceptable, the threshold for selecting profiles with better performance (*R*^2^ > 0.70) might be considered relatively low for the development of highly accurate models. Alternatively, if the relationship is indeed valid, such an outcome highlights the need for a larger sample size to improve the reliability and trustworthiness of the rule.

Since the predictive profiles derived from this study were not tested, the results should be read cautiously regarding the accuracy of real-life scenario prediction by these rules and their applicability to diverse datasets. To create generalizable prediction models, future research needs to include larger samples of FCs and (based on standard guidelines) a minimum sample size of at least 100 participants per profile to provide a more stable model. Ensuring adequate representation of each feature will contribute to increasing the generalizability and robustness of the prediction models.

## 6. Clinical Implications

The findings underscore the complexity of caregiving, highlighting the myriad challenges faced by FCs, including emotional, physical, and financial burdens. By developing predictive models, such as Profiles 1, 2, and 3, this longitudinal study offers valuable insights into the factors influencing caregiver burden perception, facilitating the early identification of FCs at risk of experiencing high burden levels. Proactive interventions guided by these risk assessment profiles have the potential to improve health outcomes for both FCs and persons living with AD, ultimately leading to more efficient and cost-effective healthcare delivery.

According to the results, distress and forgiveness at T1 and T2 were significant predictors of burden. Family stress at T1 and HRV at T2 were also relevant predictors and should be considered in the initial evaluation and subsequent monitoring. Furthermore, sociodemographic characteristics such as FC’s age and the person with AD’s age should also be considered, since they were also predictors.

An early prediction of the FC’s risk profile may lead to more expedient help, promoting proactive interventions and support strategies. This proactive approach could lead to more successful health outcomes for both the FC and the person living with AD. By identifying FCs at risk of experiencing high burden levels, healthcare professionals could implement targeted interventions tailored to the specific needs of caregivers, thereby enhancing their well-being and potentially mitigating the adverse effects of caregiving stress. As a result, FCs identified as having higher distress levels and lower forgiveness may benefit from interventions focused on stress management techniques and enhancing coping strategies. Also, FCs with better emotional well-being and higher forgiveness scores may require different types of support, such as respite care services or educational programs aimed at enhancing caregiving skills. Moreover, by reducing FC’s burden, interventions may contribute to the overall improvement in the quality of life of individuals living with AD, ultimately leading to more efficient and cost-effective healthcare delivery. Incorporating predictive models, such as those presented in Profiles 2 and 3, into clinical practice holds great promise for addressing the multifaceted challenges associated with AD caregiving and optimizing healthcare outcomes.

The present study underscores the importance of adopting a person-centered approach to care planning. By considering the unique characteristics and needs of each caregiver–care dyad, healthcare professionals may develop tailored care plans that address relational dynamics and promote positive caregiving experiences.

Although exploratory, the findings of this study hold significant potential for advancing future research in the field of caregiving for persons living with AD through the application of ML techniques. Upon validation with additional datasets, these results could offer valuable guidance, particularly in predicting caregiver burden and associated stressors for healthcare providers. Such insights may contribute to reducing the individual, societal, and economic challenges posed by the long-term caregiving of persons living with AD.

## 7. Conclusions

In conclusion, this study represents a pioneering effort in finding risk assessment profiles for caregiver burden in FCs of persons living with AD using Decision Tree algorithms. By leveraging ML techniques, complex, nonlinear relationships and interactions among caregiving variables that traditional methods might overlook were uncovered, refining caregiver burden assessment and enabling the early identification of high-risk caregivers. The use of ML techniques may inform interventions increasing the effectiveness of targeted interventions, ultimately leading to better health outcomes for caregivers and persons living with AD. The results underscore the complexity of caregiving, identifying the key predictors of burden such as distress, forgiveness, family stress, and HRV, which should be considered in clinical evaluations. Further research with larger datasets is needed to validate these findings and enhance the generalizability of predictive models.

## Figures and Tables

**Table 1:** Characterization of family caregivers and persons living with Alzheimer’s disease.

Family Caregivers					
		Min	Max	Mean	*SD*
Age		42	92	66.15	13.64
Education (years)		0	20	4.53	3.58
Duration of care (years)		0.5	9	3.98	2.20
		**Frequency**			**%**
Sex	Male		29		22.3
	Female		101		77.7
Marital status	Married		109		83.8
	Unmarried		21		16.2
Degree of kinship	Daughters		55		42.3
	Spouses		58		44.6
	Others (siblings, nieces, or close friends)		17		13.1
Co-habitation	Nuclear family		120		90.2
	Extended family		10		7.7
Caregiving hours	0–12 h		7		5.4
	13–24 h		123		94.6
First-time caregiver	Yes		92		70.8
	No		38		29.2
Chose to be the primary caregiver	Yes		103		79.2
	No		27		20.8
Presence of a secondary caregiver	Yes		71		54.6
	No		59		45.4
	**Persons living with Alzheimer’s disease**				
Continuous variables		**Min**	**Max**	**Mean**	***SD***
Age		60	101	85.19	5.97
Education		0	9	1.38	1.91
		**Frequency**			**%**
Sex	Male	44			33.8
	Female	86			66.2
Marital status	Married	66			50.8
	Unmarried	64			49.2
		**Min**	**Max**	**Mean**	***SD***
Duration of memory problems		6 months	10 years	3.93	2.29
		**Frequency**			**%**
Previous treatments for memory problems	Yes	44			33.8
	No	86			66.2
Stages of dementia (CDR)	Mild	43			33.1
	Moderate	37			28.5
	Severe	50			38.5

**Table 2:** Performance of the initial burden profile models.

	T1		T1_Base		T2		T2_Base		T1_T2		T1_T2_Base	
	*RMSE*	*R* ^2^	*RMSE*	*R* ^2^	*RMSE*	*R* ^2^	*RMSE*	*R* ^2^	*RMSE*	*R* ^2^	*RMSE*	*R* ^2^
Decision Trees	14.89	0.49	14.71	0.50	14.21	0.47	**10.52**	**0.71**	**12.21**	**0.61**	**10.78**	**0.69**
Random Forests	16.38	0.38	17.26	0.32	14.32	0.46	11.97	0.62	10.34	0.72	11.53	0.65
XGBoost	17.34	0.31	16.62	0.37	11.51	0.65	11.56	0.65	8.97	0.79	9.88	0.74
LightGBM	17.97	0.26	17.9	0.27	15.06	0.40	11.57	0.65	14.18	0.47	12.19	0.61
CatBoost	13.86	0.56	14.78	0.5	13.58	0.51	14.21	0.47	12.06	0.62	11.13	0.67

Legend: Bold font highlights the best results obtained.

## Data Availability

The data that support the findings of this study are available from the corresponding author [MGP] upon reasonable request.
